# Whole-Genome Methylation Study of Congenital Lung Malformations in Children

**DOI:** 10.3389/fonc.2021.689833

**Published:** 2021-06-28

**Authors:** Sara Patrizi, Federica Pederiva, Adamo Pio d’Adamo

**Affiliations:** ^1^ Medical, Surgical and Health Sciences Department, University of Trieste, Trieste, Italy; ^2^ Pediatric Surgery, Institute for Maternal and Child Health–IRCCS “Burlo Garofolo”, Trieste, Italy; ^3^ Laboratory of Medical Genetics, Institute for Maternal and Child Health–IRCCS “Burlo Garofolo”, Trieste, Italy

**Keywords:** congenital lung malformation, lung tumor, methylation, whole genome, children

## Abstract

**Background and Objectives:**

The treatment of asymptomatic patients with congenital pulmonary malformations (CPMs) remains controversial, partially because the relationship between congenital lung malformations and malignancy is still undefined. Change in methylation pattern is a crucial event in human cancer, including lung cancer. We therefore studied all differentially methylated regions (DMRs) in a series of CPMs in an attempt to find methylation anomalies in genes already described in association with malignancy.

**Methods:**

The DNA extracted from resected congenital lung malformations and control lung tissue was screened using Illumina MethylationEPIC arrays. Comparisons between the group of malformed samples or the malformed samples of same histology or each malformed sample and the controls and between a pleuropulmonary blastoma (PPB) and controls were performed. Moreover, each malformed sample was pairwise compared with its respective control. All differentially methylated regions (DMRs) with an adjusted p-value <0,05 were studied.

**Results:**

Every comparison highlighted a number of DMRs closed to genes involved either in cell proliferation or in embryonic development or included in the Cancer Gene Census. Their abnormal methylation had been already described in lung tumors.

**Conclusions:**

Methylation anomalies already described in lung tumors and also shared by the PPB were found in congenital lung malformations, regardless the histology. The presence of methylation abnormalities is suggestive of a correlation between congenital lung malformations and some step of malignant transformation.

## Introduction

There is a general consensus that symptomatic congenital pulmonary malformations (CPMs) should be removed surgically. However, the treatment of asymptomatic patients remains controversial (1). Some authors recommend prophylactic pulmonary resection to avoid the long-term risk of pulmonary recurrent infections, pneumothorax, or development of lung malignancy (1, 2), while others suggest a conservative approach based on the observation of the patient (1, 3).

The relationship between congenital lung malformations and malignancy remains undefined and continues to be a critical consideration in surgical decision making. Hartman and Stochat reported that 4% of pulmonary malignant tumors were associated with congenital cystic malformations. Tumors developing within these malformations included rhabdomyosarcoma, pleuropulmonary blastoma (PPB), adenocarcinoma, squamous cell carcinoma, and mesenchymoma (4). Later, Ozcan et al. reported 29 cases of primary rhabdomyosarcoma, 15 of which arose in a preexisting congenital lung malformations (5). Nasr et al. (6) found 2% of association between PPB and congenital pulmonary malformations. Recently, in a systematic review (7) we highlighted 168 cases, 76 children and 92 adults, in whom a lung tumor was found in association with a CPM. We concluded that all histological types of CPMs could be associated with malignant lung lesions and that the malignant transformation could happen at any age.

One hallmark of cancer cells is their completely different methylation pattern. In many malignant tumors, the levels of methylation are decreased, while promoter regions of important regulatory and tumor suppressor genes are hypermethylated and therefore silenced. Hypermethylation associated with tumor suppressor genes is uncommon in normal cells. However, it is widely represented in cancer cells (8, 9). Abnormal DNA hypomethylation has been demonstrated to also play an important role in tumor development, both increasing genome instability (10) and activating the transcription of oncogenes that are normally silenced (11).

The aim of this study was to investigate the possible biologic relationship between congenital pulmonary malformations and lung tumors. Using Illumina MethylationEPIC array analysis that is easy to use, time efficient, and cost effective technique (12), we studied all differentially methylated regions (DMRs) in a series of congenital lung malformations in an attempt to find methylation anomalies in genes already described in association with malignancy.

## Material and Methods

All congenital lung malformations resected at the Institute for Maternal and Child Health–IRCCS “Burlo Garofolo” (Trieste, Italy) from January 2010 to January 2019 were assessed. After approval by the Institutional Ethical Committee, the medical records of the patients were analyzed.

Lung biopsies from the resected malformed lobes were snap-frozen and stored at −80°C. Samples used as control included biopsies from macroscopically normal lung tissue adjacent to the malformation of seven patients, three with intralobar sequestration (ILS) and four with congenital pulmonary airway malformation type 2 (CPAM2) and from the lung of a patient who was thought to have a congenital malformation until histological analysis proved the tissue to be normal. Lung biopsies from a patient with PPB were also analyzed. We were not able to recruit other patients with the same tumor firstly because of its rarity and, secondly, because it is frequently mistaken with other diagnostic entities and formalin-fixed and paraffin-embedded. This technique of storage could lower the quality of the DNA, and therefore the comparison with higher quality DNA from fresh frozen tissues could generate artifacts. Moreover, pediatric patients with lung malignancies are also absent from public databases such as The Cancer Genome Atlas (TCGA) (13) or ENCODE (14). It is also not possible to compare methylation data from adult lung malignancies to our pediatric malformations because the methylation pattern changes drastically with age (15).

Genomic DNA was extracted from lung tissue samples (16), and its concentration was measured with Qubit dsDNA Broad Range Assay Kit (Thermo Fisher Scientific). Of each DNA sample, 1 μg was bisulfite-converted with EZ DNA Methylation Kit and screened using MethylationEPIC Beadchips according to the manufacturer’s instructions (Zymo Research and Illumina Inc. respectively). Raw methylation data were analyzed with R version 3.6.2 (2019-12-12), using package ChAMP (17) (Chip Analysis Methylation Pipeline) version 2.16.1 in Rstudio.

Standard parameters of function champ.load were used to load and filter the dataset. After normalization with BMIQ method, the effect of two confounding variables (age of the samples and beadchip of origin) was removed using function removeBatchEffect from package Limma. All analyses were performed on the corrected dataset.

Significant differentially methylated regions (DMRs) were then calculated using the Bumphunter algorithm applying 1,000 permutations to each comparison, and the p-value was adjusted for multiple testing.

In our first analysis we compared the cohort of malformed samples with controls, looking for changes to the methylation pattern common to all malformations, regardless of histology. Then, we compared groups of the same histology with controls to identify any methylation signatures shared by cases of the same histological profile. Finally, we compared each individual malformed sample with the group of controls for individual differences. For eight samples that had a control tissue from the same patient available, we also performed a pairwise DMR analysis, using the standard parameters of R package DMRforPairs (18). Furthermore, the methylation profile of the malformed samples was compared with that of the single tumor sample to highlight any similarities.

At each comparison, the identified DMRs were classified according to their location, mapping to either the gene body, the TSS (transcription start site), the 5′ UTR (untranslated region) or the 3′ UTR of protein-coding genes of interest (that we refer to in the manuscript as GOI); or mapping to an intergenic region that according to the ENCODE database contains a candidate cis-regulatory element (ccRE) close to a GOI. These genes were either included in the Cancer Gene Census (CGC, Cosmic (19) or implicated in embryonic development or involved in cell proliferation (both according to the AmiGO 2 database (20), and the overlap between the three groups of genes was also taken into consideration.

Raw data was uploaded to Gene Expression Omnibus as dataset GSE174625.

## Results

Eighteen patients, nine girls and nine boys, all Caucasian except for one of African ethnicity, who underwent lung resection for congenital lung malformations, were considered. Thirteen of them had prenatal diagnosis. Ten patients remained asymptomatic, while eight had different degrees of respiratory infection. Six patients had intralobar sequestration (ILS), nine had congenital pulmonary airway malformations (CPAM) associated with extralobar sequestration (ELS) in two cases. Clinical data and histology are summarized in **Table 1**.

**Table 1:** Clinical features and histopathology of patients with congenital lung malformations.

Patient n	Sex	Prenatal diagnosis	Age at surgery (months)	Symptoms	Surgical procedure	Histology
1	F	no	21	Respiratory infections	Left lower segmentectomy	ILS
2	F	yes	5	Asymptomatic	Left lower lobectomy	ILS
3	M	no	10	Respiratory infections	Right lower lobectomy	ILS
4	F	yes	5	Respiratory infections	Left upper lobectomy	CPAM 2
5	M	yes	5	Asymptomatic	Right middle lobectomy	CPAM 3
6	F	yes	6	Asymptomatic	Right lower lobectomy	CPAM 3
7	M	yes	60	Respiratory infections	Right middle lobectomy	CPAM 3
8	M	no	108	Right lung pneumonia	Right upper lobectomy	CPAM 1
9	F	yes	57	Respiratory infections	Left lower lobectomy	ILS
10 (A + B)	M	no	120	Pneumonias	Left lower lobectomy + ELS resection	CPAM 2 + ELS
11	F	yes	7	Asymptomatic	Left lower lobectomy	CPAM 2
12	F	yes	9	Asymptomatic	Left lower lobectomy	ILS
13	M	no	140	Pneumonias	Left lower lobectomy	CPAM 2
14	F	yes	14	Asymptomatic	Left lower lobectomy	CPAM 2
15	F	yes	8	Asymptomatic	Right middle lobectomy	CPAM 1
16	M	yes	9	Asymptomatic	Left upper lobectomy	CPAM 2
17	M	yes	9	Asymptomatic	Right lower lobectomy	ILS
18 (A + B)	M	yes	10	Asymptomatic	Right upper lobectomy + ELS resection	CPAM 2 + ELS

**Figure 1:**
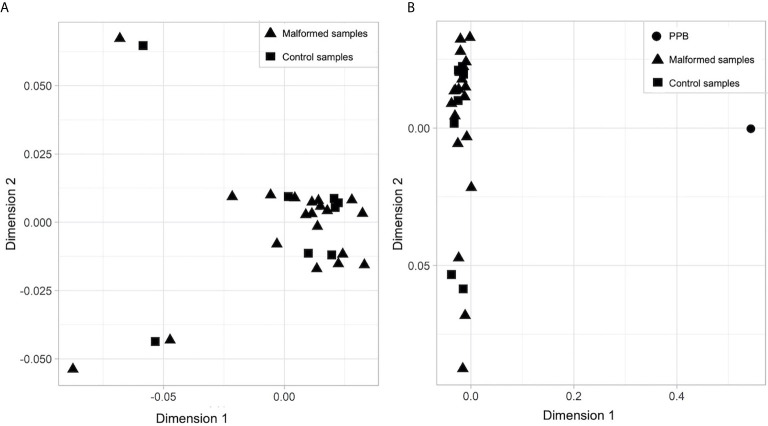
Multidimensional scaling (MDS) plot of **(A)** malformed and control samples; **(B)** malformed, control and pleuropulmonary blastoma samples. In each plot, the X axis represents Dimension 1 and the Y axis Dimension 2 of the MDS statistical analysis, which better expresses the mathematical distance between the samples.

No clear separation between the malformed and the control samples was found on a multidimensional scaling (MDS) plot representing the similarity of all samples based on the signal of the top 1,000 most variable probes. They form a homogeneous group (**Figure 1A**) completely separated from the PPB sample (**Figure 1B**).

**Figure 2:**
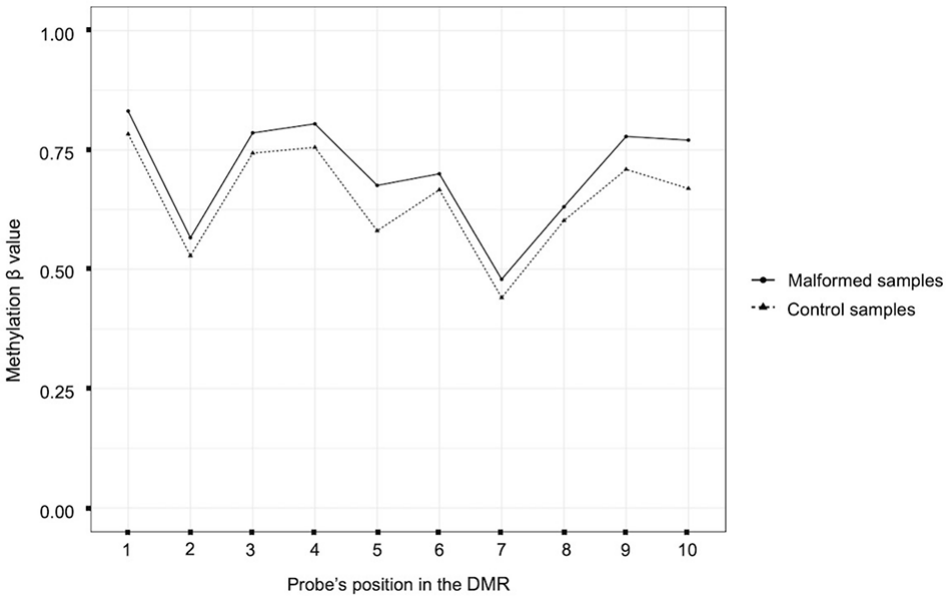
Plot of the beta methylation values (Y axis) of each probe (X axis) of the DMR located near the gene ZFP57. The beta values range from 1 (completely methylated) to 0 (completely unmethylated). The position of the probes inside the DMR is ordered according to their genomic position.

The comparison between all the malformed samples and the controls identified 10 statistically significant DMRs (with and adjusted p-value < 0,05). Among them, one involved a ccRE located in an intergenic region near exon 1 of the gene ZFP57. In this region the mean methylation beta value was slightly higher in the malformed group than in the control group (**Figure 2**).

**Figure 3:**
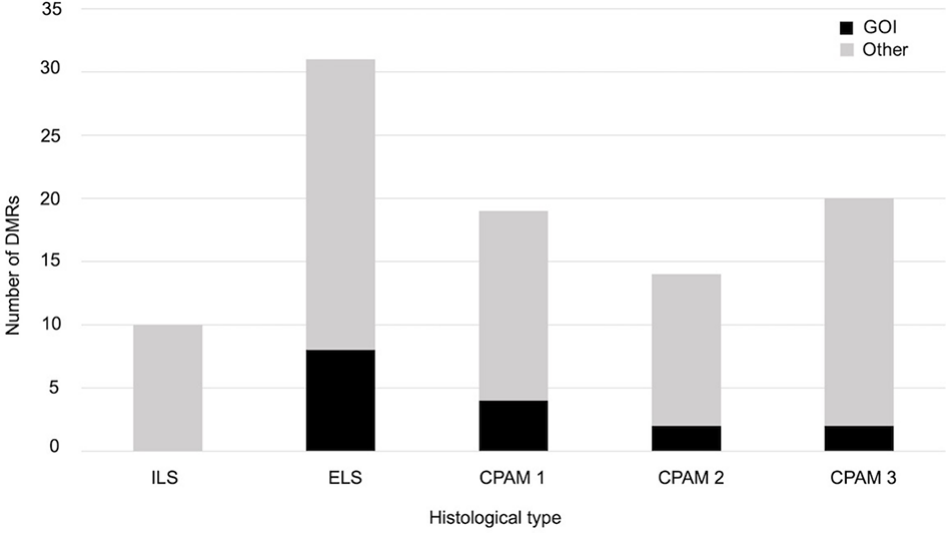
Number of significant DMRs (adjusted p-value < 0,05), close to GOI (black) and to different genes (gray), identified when the cases of same histology were compared with controls.

We also checked whether samples with the same histology have DMRs in common and that differ from controls. We found 10 significant DMRs in the ILS samples, none of which was close to a GOI. In the other histological type there were no DMRs near genes involved in cancer, but some were close to other GOIs, related to cell proliferation or embryonic development: in particular, seven out of 31 in the ELS samples, four out of 19 in the CPAM1 samples, two out of 14 in the CPAM2 samples, and two out of 20 in the CPAM3 samples (**Figure 3** and **Table 2**).

**Table 2:** GOI close to significant DMRs elicited by the comparison between cases of same histology with controls.

Histology	Gene(s) in or near the DMR	GOI
ELS	HOXB1	development
	HOXD4	development
	CTNNA1	proliferation
	NR2F2	proliferation and development
	HSF4	proliferation
	MEIS1	proliferation
	PTN	proliferation
CPAM 1	PLD6	development
	TXNRD1	proliferation and development
	S100A13	proliferation
	MSX2	proliferation and development
CPAM 2	ZFP57	development
	MEIS1	proliferation
CPAM 3	MSX2	proliferation and development
	PITX2, ENPEP	proliferation

**Figure 4:**
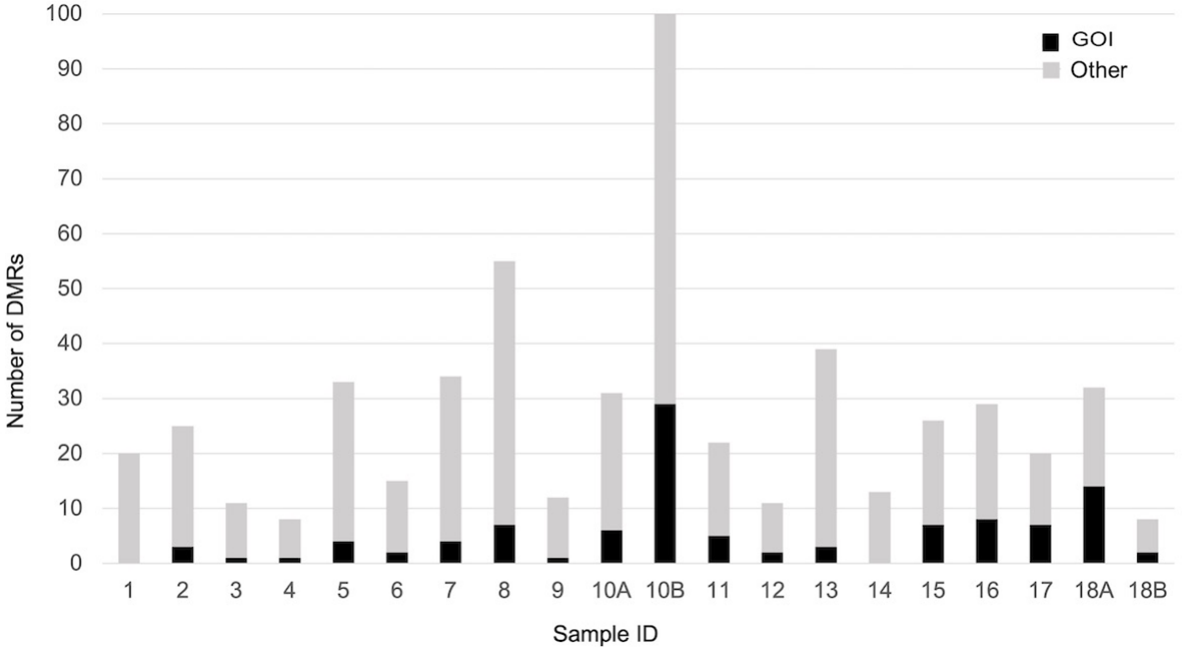
Number of significant DMRs (adjusted p-value < 0,05), close to GOI (black) and to different genes (gray), identified comparing each malformed case with controls.

**Figure 5:**
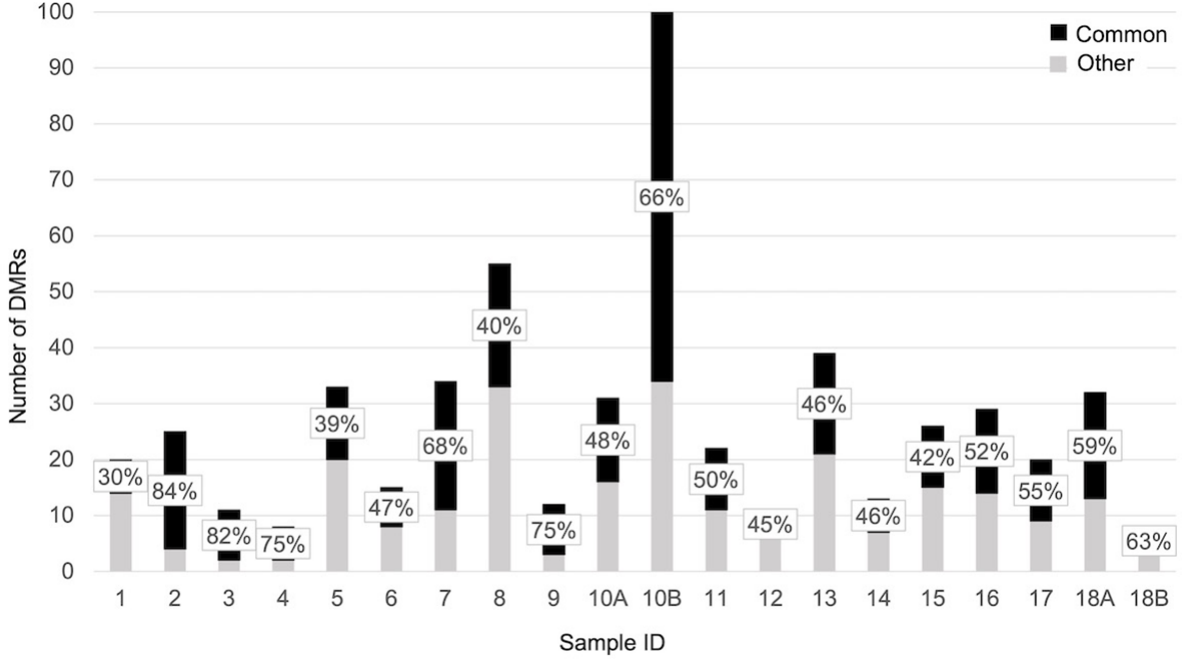
Bar plot representing the percentage of significant DMRs (adjusted p-value < 0,05) shared by each sample with the PPB.

When each malformed sample was compared with the controls, the number of DMRs ranged from eight, in samples 4 and 18B, to 100 in sample 10B (**Figure 4**). Nine of them were repeated at least three times and were localized close to genes which act in cell proliferation or in embryonic development or are included in the CGC (**Table 3**). The percentage of DMRs that each sample had in common with the PPB sample ranges from 30% in sample 1 to 84% in sample 2 (**Figure 5**).

**Table 3:** GOI close to significant DMRs repeated at least three times when each malformed sample was compared to the controls.

Patient n	Gene(s) in or near the DMR	GOI
2	HOXB1	development
	SIX1, SIX4, MNAT1	cancer, proliferation and development
5	ZFP57	development
	IRAK4	proliferation
	MSX2	development
6	ZFP57	development
	MSX2	proliferation and development
7	ZFP57	development
	IRAK4	proliferation
	MSX2	proliferation and development
	NR2F2	proliferation and development
	MEIS1	proliferation
	HOXB1	development
	HOXD4	development
11	ZFP57	development
12	ZFP57	proliferation
	IRAK4	proliferation
13	ZFP57	development
	IRAK4	proliferation
17	ZFP57	development
	MMP2, IRX5	proliferation and development
	HOXD4	development
	MEIS1	proliferation
	IRAK4	proliferation
	SIX1, SIX4, MNAT1	cancer, proliferation and development
16	HOXD4	development
	ZFP57	development
18a	ZFP57	development
	HOXB1	development
	HOXD4	development
	NR2F2	proliferation and development
	MEIS1	proliferation
	MMP2, IRX5	proliferation and development
	SIX1, SIX4, MNAT1	cancer, proliferation and development
18b	ZFP57	development
	HOXD4	development

The pairwise DMR analysis, limited to the samples that had a control tissue from the same patient available, identified statistically significant DMRs in three out of eight samples: 23 in sample 10B, four in sample 12, and two in sample 17. The number of DMRs near the genes of interest was respectively thirteen, two, and one (**Table 4**).

**Table 4:** GOI close to significant DMRs elicited by the pairwise comparison between each malformed sample and the corresponding control tissue.

Patient n	Gene(s) in or near the DMR	GOI
10B	HOXA3	development
	CTNNA1	proliferation
	CTSZ	proliferation
	NR2F2	proliferation and development
	GPR37L1	proliferation
	IGF2BP1	proliferation
	TGFB1I1	proliferation and development
	HOXC6	development
	TSPAN32	proliferation
	S100A13	proliferation
	BRD2	development
	HOXB1	development
12	SMAD6	proliferation and development
	FOXP1	proliferation and cancer
17	HOXD3–HOXD4	development

## Discussion

Congenital pulmonary malformations are developmental abnormalities of the lung occurring in approximately one to 4.2 per 10,000 births (21). Postnatal presentation varies from severe respiratory distress to complete lack of symptoms (22). Although all authors agree that surgical resection is the standard of care for symptomatic cases, the management of asymptomatic lesions remains controversial. The main reason for recommending elective surgery is late development of complications, including the highly debated malignancy. The relationship between congenital lung malformations and malignancy remains unknown and continues to be a critical consideration in surgical decision making. Some studies have addressed this issue in an attempt to find premalignant characteristics in congenital lung malformations.

Vargas and colleagues analyzed the karyotype and looked for p53 mutations in congenital cystic adenomatoid malformations. As they did not find cytogenetic abnormalities in congenital cystic adenomatoid malformations by conventional karyotype analysis, they concluded that congenital lung anomalies were non-neoplastic (23). Later, Rossi and colleagues found expression of mucins and K-RAS mutations in mucinogenic proliferations of congenital pulmonary airway malformations and concluded that these findings supported the neoplastic nature of the malformations (24). Recently, Hsu et al. (25) described in the blood of 19 cases of CPAM several damaging variants in genes involved in lung carcinoma.

The aim of this study was to look for further insight into the biologic relationship between congenital pulmonary malformations and lung tumors. As the change in methylation pattern is a pivotal event in human cancer including lung cancer, in this study we focused on DNA methylation in a series of congenital lung malformations.

Firstly, we compared all the malformed samples with the controls identifying, among ten, only one DMR of interest, involving a candidate cis-regulatory element (ccRE) and located near exon 1 of the gene ZFP57, which acts in genome imprinting, regulation of gene expression, and cell signaling. It has recently been hypothesized that ZFP57 is a potential susceptibility gene for lung cancer development through the increase of IGF2 expression (26).

The analysis did not uncover a common methylation pattern since the significant DMRs were very few. This was not surprising, as the malformed samples were a very heterogeneous group, and the individual differences in the methylation patterns of each malformed sample, combined with the likely cellular heterogeneity of the samples (that was beyond the control of the investigators), masked the differences between the two groups.

Looking for a common methylation signature among the cases of CPMs, we then compared the cases of same histology with controls. The results highlighted seven DMRs in the ELS close to genes mostly involved in embryonic development (at the TSS of HOXB1, HOXD4) and cell proliferation (at the TSS of CTNNA1, NR2F2, HSF4, MEIS1, and at the gene body of PTN). Both HOXB1 and HOXD4 were abnormally expressed in lung tumors. HOXB1 was described down-regulated in lung cancer tissue (27) and, similarly, we found the DMR close to this gene was hypermethylated. HOXB1 is an anti-tumor gene acting by inhibiting the expression of survival oncogenic genes (28); its decreased expression had been correlated with cell invasion and proliferation and inhibited apoptosis in glioma (29). HOXD4 was reported up-regulated in lung squamous cell carcinoma resulting in more aggressive invasiveness of lung cancer cells (30). In our samples of ELS HOXD4 was hypermethylated in an opposite fashion compared to those in the literature. Either CTNNA1 (31) or NF2F2 (32) has been found significantly down-regulated in lung cancer. The DMR close to CTNNA1 was hypermethylated in our samples in line with previous report of the literature. NF2F2 was also known to play a role in angiogenesis and development and to contribute to transform a non-invasive lung cancer in an invasive one through its expression (33). MEIS1 might limit the proliferation of non-small-cell lung adenocarcinoma (34) and was described methylated in squamous cell carcinomas (35). PTN is highly expressed in embryonic and postnatal development, while it is quite down-regulated in adult life; however, it is strongly expressed in lung tumor and other types of cancer (36). Hypermethylation was found in our samples for the DMR close to MEIS1 and PTN.

Four DMRs were found in CPAM1 cases close to genes related to cell proliferation or embryonic development (at the TSS of PLD6, S100A13; MXS2, and at the 5′ UTR of TXNRD1). S100A13’s overexpression was associated with intratumoral angiogenesis and more aggressive invasive phenotype in non-small-cell lung cancer (37). In our samples the DMR was hypermethylated differently from those in the literature. MSX2 is a regulator of embryonic development, and it is involved in pancreatic and breast cancer, but recently it has been found that it plays a role also in lung adenocarcinoma (38). The DMR was hypermethylated in our samples.

We found two DMRs in CPAM2 cases, close to the already discussed intergenic region near ZFP57, acting in embryonic development, and the gene body of MEIS1, whose closed DMR was hypomethylated differently from the mechanism in literature.

Finally, CPAM3 cases presented two DMRs, one close to the TSS of MSX2 which was this time hypomethylated, and the other in an intergenic region involving a ccRE near two genes involved in proliferation that was hypomethylated as well. The first gene near the second DMR was PITX2, whose low methylation correlated with higher risk of lung cancer progression (39), and the other was ENPEP that was under-expressed in lung adenocarcinoma (40).

One more time, the heterogenicity of the malformed samples likely prevented to highlight a common methylation signature. However, the presence of methylation abnormalities in genes already described in association with malignancy is suggestive, especially considering their role in lung tumors.

Due to the heterogeneity in our malformed samples, we also checked for individual differences by comparing each sample with the control group. It yielded a wide range of DMRs, but nine of them recurring at least three times: three including one ccRE each, near the genes ZFP57, SIX1-SIX4-MNAT1, and MMP2-IRX5; four at the TSS of genes HOXB1, HOXD4, NR2F2 and MSX2; one at the 5′ UTR of IRAK4, and one at the gene body of MEIS1. ZFP57, HOXB1, HOXD4, NR2F2, MEIS1, and MSX2 were already described when the cases of same histology were compared with controls. Interestingly, the DMR close to HOXB1 was found hypomethylated and the one close to HOXD4 hypermethylated, in the opposite way to the literature. SIX1 and SIX4 were associated with increased risk of tumorigenesis when their expression was increased. SIX4 controlled the expression of oncogenes, and it correlated with higher stages of the tumor, poor survival in NSCLC, and worse rate of relapses in lung adenocarcinoma (41). MNAT1 might have a role in promoting the development of NSCLC (42). MMP2 mRNA and protein levels were found increased in NSCLC (43).

The pairwise DMR analysis found statistically significant DMRs in three out of eight samples, when comparing each one of them with the corresponding control tissue from the same patient. The number of DMRs near to genes of interest was 12 out of 23 in one of the ELS (sample 10B), two out of four in an ILS (sample 12), and one out of two in another ILS (sample 17). In the ELS, the DMRs of interest were located at the gene body or the TSS of genes involved in embryo development (HOXA3, HOXC6, HOXB1, TGFB1I1, BRD2), cell proliferation (CTNNA1, CTSZ, GPR37L1, S100A13, TSPAN32, FOXP2), or both processes (IGF2BP1, NR2F2). In one of the ILS (sample 12), one DMR of interest was hypomethylated with respect to the control tissue, and located at the gene body of SMAD6 that has been reported as a “master regulator” of lung adenocarcinoma, for which both oncogenic and tumor suppressing activities have been proposed (44). The second DMR of interest for the same sample was a hypermethylated region at the gene body of FOXP1, a transcription factor involved in cell proliferation and included in the CGC that according to Sheng et al (45), prevented the growth of lung adenocarcinoma cells by the suppression of chemokine signaling pathways. The DMR of interest in the other ILS (sample 17) was located at the TSS of HOXD4, a gene that has been discussed earlier, and it was less methylated in the malformed than in the control tissue, contrarily to our previous findings but in concordance with the literature.

Both types of single sample analyses have strengths and weaknesses. Comparison of one sample to the entire control group allowed the analysis of samples that do not have a control tissue from the same patient, but it could introduce artifacts due to inter-patient variability of the methylation profile. The pairwise analysis, on the other hand, was possible in this case for a smaller number of samples, but it excluded any underlying methylation alterations not strictly related to the malformation, thus producing more specific results.

Finally, we compared the PPB sample with the controls and considered to discuss, among all, the DMRs in common with the malformed samples, mapping to the 3′ UTR of CACNA1C, the 5′ UTR of HOXA5, the TSS of CTSZ, ESRP2, HAND2, HOXA2, HOXA3, MAGI2, TWIST1, ccREs near MMP2, MSX1-OTX1-RUNX1, TBX3–TBX5, and the gene body of SH3PXD2A and WT1).

CACNA1C was found down-regulated in lung cancer (46). CTSZ was involved in promoting NSCLC cell migration and invasion (47). ESRP2 maintained the epithelial phenotype, avoiding the epithelial to mesenchymal transition that contributed to metastases. In NSCLC it was inhibited (48). HAND2 was found either hypermethylated or hypomethylated in different stages of lung adenocarcinoma (49). HOXA2, HOXA3, and HOXA5 have been recognized as target for DNA methylation in lung cancer, and they promoted carcinogenesis, but also acted as tumor-suppressor factors (50). MAGI2 was reported to act as an anti-tumor in hepatocellular cancer and breast cancer; its down-regulation has been demonstrated in NSCLC (51). CpG islands associated with MSX1 and OTX1 were methylated in the majority of lung squamous cell carcinomas, while the ones associated with RUNX1 were methylated in more than 80% of lung adenocarcinomas, being well known that hypermethylation of CpG islands is a signature of malignant progression (35). SH3PXD2A’s increased expression in lung adenocarcinoma directly correlated with metastasis and worse prognosis for the patient (52). TBX5 and TBX3 were highly expressed in normal lungs, but significantly suppressed in lung adenocarcinoma (53). TWIST1 expression was found increased in lung cancer tissue (54). WT1 was described as oncogene in lung cancer, among other malignancies (55).

The DMRs of interest were spread throughout the cases, and we were not able to find a recurrent pattern of abnormalities in the different types of congenital lung malformations.

It has been demonstrated that lung cancer develops through the acquisition of alterations in oncogenes and tumor suppressor genes. Prolonged exposure to carcinogens that interact with various genetic susceptibility and/or resistance factors contributes to the accumulation of genomic alterations, including nucleotide substitutions, small insertions and deletions, and chromosomal rearrangements in human lung cancer (56). This mechanism of action may explain the cases of association between congenital pulmonary malformations, sometimes asymptomatic for many years, and lung tumors described in literature (7), in which the genetic susceptibility together with the progressive exposure to carcinogens might trigger the development of malignancy.

This study has highlighted some key points. First, methylation anomalies already described in lung tumors could be found in samples of congenital lung malformations, regardless the histology, both when compared to the control group and when compared to a specific control tissue. Second, some methylation anomalies of the congenital lung malformations were shared by the PPB. Third, it seems unlikely that the presence of methylation abnormalities, which have been reported in association with lung tumors, could be considered a casual event in congenital lung malformations, in which malignant transformation has been described (7). Pulmonary malformations are essentially due to a dysregulation of cell proliferation which, during organogenesis, creates an abnormal development of some areas of the lower respiratory tract. Our study indicates that, at least in part, this dysregulation is caused by some genes which, due to their role in the cell cycle, are also involved in some stages of tumor development.

Our study, however, had some limits that should be acknowledged, such as the small number of samples of congenital lung malformations included and their heterogenicity. Moreover, the difficulty in finding proper control samples has limited their number. Another limitation is the lack of material from pediatric adenocarcinoma of the lung, which is the second tumor for frequency that has been found in association with CPMs in children (7), and the impossibility to compare the methylation of pediatric CPMs to the available adult adenocarcinoma samples due to the age-related changes in the methylation pattern. To our knowledge, this study represents the first attempt to address the methylation anomalies in pediatric congenital lung malformations using a whole genome approach. We have also described some methylation changes which, in some genes, appeared to have the opposite sign to what is described in the literature regarding their expression. These results, however, should not be discarded, because the relationship between DNA methylation and gene expression could be more complicated than previously understood [for example, it can have different effects according to its position with respect to the gene (57)], and DNA methylation is only one of the factors that regulate the expression of a gene. Thus, since we did not study the gene expression in our samples, we cannot be certain that hypomethylation corresponds to hyperexpression or *vice versa*.

## Conclusion

Methylation anomalies already described in lung tumors and also shared by the PPB were found in congenital lung malformations, regardless the histology. This is suggestive of a correlation between congenital lung malformations and some step of malignant transformation.

More detailed analysis of genetic and epigenetic interactions as well as functional interactions among genes altered in congenital pulmonary malformations will further provide insights into the molecular mechanism of lung carcinogenesis.

## Data Availability Statement

The original contributions presented in the study are publicly available. This data can be found here https://www.ncbi.nlm.nih.gov/geo/query/acc.cgi?acc=GSE174625.

## Ethics Statement

The studies involving human participants were reviewed and approved by IRCCS Burlo Garofolo. Written informed consent to participate in this study was provided by the participants’ legal guardian/next of kin.

## Author Contributions

FP and AD’A conceptualized and designed the study, drafted the initial manuscript, and reviewed and revised the manuscript. SP designed the data collection instruments, collected data, carried out the initial analyses, and reviewed and revised the manuscript. All authors contributed to the article and approved the submitted version.

## Funding

This study was supported by the IRCCS “Burlo Garofolo” (RC 22/11). The funder/sponsor did not participate in the work.

## Conflict of Interest

The authors declare that the research was conducted in the absence of any commercial or financial relationships that could be construed as a potential conflict of interest.

## Abbreviations

ccRE, cis-regulatory element; CGC, Cancer Gene Census; CPAM1, 2, 3, congenital pulmonary airway malformation types 1, 2, 3; CPM, congenital pulmonary malformation; DMR, differentially methylated region; DNA, deoxyribonucleic acid; ELS, extralobar sequestration; PPB, pleuropulmonary blastoma; GOI, genes of interest; ILS, intralobar sequestration; MDS, multidimensional scaling; TCGA, The Cancer Genome Atlas; TSS, transcription start site; UTR, untranslated region.
